# Chronic NO Restriction in Hypertensive Rats Increases Abdominal but Not Thoracic Aortic Intrinsic Stiffness via an Augmentation in Profibrotic Materials

**DOI:** 10.1155/2019/8070198

**Published:** 2019-03-19

**Authors:** George Lindesay, Yvonnick Bézie, Christophe Ragonnet, Véronique Duchatelle, Marc Isabelle, Nicole Villeneuve, Christine Vayssettes-Courchay

**Affiliations:** ^1^Servier Research Institute, Cardiovascular Discovery Research Unit Suresnes, France; ^2^Department of Pharmacy, Groupe Hospitalier Paris Saint-Joseph, 185 Rue Raymond Losserand, 75014 Paris, France; ^3^Department of Pathology, Groupe Hospitalier Paris Saint-Joseph, Paris, France

## Abstract

The spontaneously hypertensive rat model with reduced NO synthesis (SHRLN) shares features with aging and hypertension in humans, among other a severe aortic stiffening. The present* in vivo* study aimed to compare thoracic (TA) and abdominal (AA) aortic stiffness in the SHRLN (treated 5 weeks with L-NAME), SHR, and normotensive Wistar Kyoto (WKY). Dynamic properties of TA and AA were measured in the same rats, using echotracking recording of aortic diameter coupled with blood pressure (BP). Measurements were performed first at operating BP and then after BP reduction in hypertensive rats, thus in isobaric conditions. Histological staining and immunohistochemistry were used for structural analysis at both sites. At operating pressure, BP and pulse pressure (PP) were higher in SHRLN compared with SHR. Stiffness index was also increased and distensibility decreased in both TA and AA in SHRLN. At WKY-matched blood pressure, isobaric AA parameters remained specifically altered in SHRLN, whereas TA recovered to values identical to WKYs. Collagen, fibronectin, *α*5-selectin, and FAK were increased in SHRLN compared with SHR or WKY. Nevertheless, only the strong accumulations of fibronectin and collagen at the AA site in SHRLN were associated with intrinsic stiffening. In conclusion, we confirm that NO restriction associated with hypertension induces a severe pathological phenotype and shows that L-NAME induced stiffening is more pronounced in AA than in TA as a result of greater fibrosis.

## 1. Introduction

The stiffening of large central arteries occurs naturally with aging. The reduction of aortic compliance leads to downstream damage to organs which receive high flow with low impedance such as the brain [1] and kidneys [2]. The concept of early vascular aging [3] describes how age-dependent arterial stiffening is accelerated with hypertension, metabolic disorders [3–7], kidney disease, and salt consumption [8–10]. A common feature of these factors which accelerate vascular aging is the reduction of nitric oxide (NO) availability [11], generally brought about by endothelial dysfunction and oxidative stress. This loss of NO availability seems to both precede and be involved in a vicious cycle of vascular remodeling which leads to increased arterial stiffness. In order to study the deleterious effect of NO bioavailability reduction, we developed a model of spontaneously hypertensive rats (SHR) treated with a moderate dose of nitric oxide synthase inhibitor L-nitroarginine methyl ester (L-NAME) [12, 13]. We previously showed that this model leads to increased hypertension and an increase in aortic stiffness associated with aortic remodeling and fibrosis [13, 14]. This model also develops cardiac ventricular hypertrophy as well as kidney damage and increased short-term blood pressure variability [15]. Importantly, these induced features, which are characteristic of cardiovascular disease, were similar to those observed in old SHR [14]. Altogether, this experimental model of severe hypertension seems relevant to investigate the mechanisms involved in hypertensive human pathology and also to help understand the major role of NO loss in pathophysiological conditions.

Recently, we investigated the role of an increased salt diet in hypertensive rat on aortic stiffness [10]. We observed that the abdominal aorta (AA) was more severely stiffened than the thoracic aorta (TA), without identification of any obvious structural changes in the aortic wall composition.

In the present study we aimed to evaluate thoracic and abdominal arterial compliance and composition in another experimental model of hypertensive aortic stiffness. For that purpose, we administered L-NAME in spontaneously hypertensive rats (SHR) over 5 weeks and studied aortic compliance using echotracking evaluation of pressure-independent stiffening [7, 13] and the aortic tissue composition via immunohistochemistry [10, 15] at both aortic thoracic and abdominal sites.

## 2. Methods

### 2.1. Animals

This study was conducted in accordance with European Community Guidelines for the use of experimental animals and was approved by the ethical committee on Animal Experiments of the Servier Research Institute. All animals were provided by CERJ (France). Three groups were compared: Wistar Kyoto (WKY) rats (n=6), SHR (n=6), and SHR given N-nitro-L-arginine methyl ester (L-NAME, Sigma) at 2 mg/kg in drinking water for 5 weeks, from 15 to 20 weeks of age (SHRLN) (n=8). Water consumption and body weight were measured every 3 days and L-NAME concentration was adjusted to maintain 2 mg/kg/day. The animals were housed 2 per cage in a temperature controlled room (20-21°C) with a 12/12 hour light/dark cycle.

### 2.2. Hemodynamic Measurements

Rats were anaesthetized with an intraperitoneal injection of sodium pentobarbital (50mg/kg i.p. for induction, maintained with 5mg/kg/h i.v. to obtain a stable level of anaesthesia). The jugular vein was cannulated for constant administration of anesthetic and the penile vein was cannulated for administration of other drugs. The trachea was cannulated and ventilation was maintained with a pressure controlled respirator (Hallowell EMC, TEM) at a frequency of 60-70 cycles per minute and a pressure of 9-12 cmH_2_O. Body temperature was maintained at 37°C with a homeothermic blanket (Harvard) connected to a rectal probe.

A microtip pressure catheter (Millar 1.2F) was inserted into the aorta via the right femoral artery. The blood pressure signal was visualized and analyzed with Biopac 4.2 Acknowledge acquisition and analysis system (CEROM). The aortic diameter was simultaneously measured as previously described [7, 10] with an ultrasound probe (L10-5 40mm 10Mhz) placed on the shaved skin on the side of the animal and was manipulated until a clear B-mode image of the thoracic aorta and the intra-arterial catheter was seen. A section of artery adjacent to the catheter was selected and subsequently analyzed in M-mode. Vessel wall tracking technology (Art.Lab, Esaote, Netherlands) was used to measure the changes in arterial diameter for 6 seconds (~30 cardiac cycles). The blood pressure signal was split and sent to a second computer containing Art.Lab to allow for blood pressure and diameter synchronization. These data were subsequently analyzed using a specialized Matlab (Mathworks) program which integrates blood pressure and diameter data and therefore allowed for arterial stiffness measurements. A more detailed description of both data acquisition via Art.Lab [16] and specific analyses within Matlab [13] has previously been published.

Thoracic measurements were made at the lowest part of the TA, above renal artery bifurcation and below diaphragm and abdominal aorta measurements were made at the lowest abdominal site.

The catheter was inserted first into the TA via the right femoral artery and measurements were made in the TA at baseline blood pressure. Then the catheter was withdrawn and placed in the AA just above the iliac bifurcation. The ultrasound was also moved to obtain an image of the catheter within the AA. For the SHR group, after the baseline recordings, a second series of measurements were taken within the AA when blood pressure was reduced using an injection of clonidine (3*μ*g/kg i.v.) to achieve the same blood pressure as the normotensive WKY rats; the effect of clonidine lasts approximately 10 min. In the SHRLN group two measurements were performed during clonidine effect, one matched BP with that of SHR and one matched BP with that of WKY. Following these measurements, the catheter and ultrasound probe were repositioned into and above the TA to obtain measurements at the baseline blood pressure of the normotensive rats. The changes induced by the reduction in BP represent the pressure dependent stiffness and the difference remaining versus normotensive animals after BP reduction represents the pressure-independent stiffening, i.e., intrinsic stiffening.

The parameters automatically calculated to determine the dynamic properties of the aortic wall were as follows: mean diameter (*D*) and diastolic diameter (d*D*); mean distension (in *μ*m); compliance (Δ*A*/ΔP) in mm^2^/kPa, where *A* is the transsectional area of the vessel calculated from the diameter and P is pressure; distensibility (Δ*A*/ΔP X*A*) in 1/kPa; and stiffness index {[d*D* ln(SAP/DAP)]/(s*D-*d*D*)},where s*D *is systolic diameter, SAP is systolic arterial pressure, and DAP is diastolic arterial pressure. Aortic distension was expressed in percent vs. diastolic diameter (Δ*D *X100/d*D*). The local pulse wave velocity (PWV) is calculated from √^−^(A/ΔP/*ρ*ΔA), *ρ*= blood viscosity. Δ represents the systolodiastolic difference during the cardiac cycle.

Additionally we analyzed as previously described [13] the pressure wave and diameter distension wave by recording both signals at 980 Hz (1.02 ms intervals) and averaging them over around 30 cardiac cycles. Pulse pressure (pp) and the distension (d) wave transformed in percent/diastolic diameter were then quantified by the area under the curve (AUCd and AUCpp respectively) corrected by 1/heart rate to avoid heart cycle duration influence. The time recording and AUC/ms were then possibly averaged within groups. A distensibility index was calculated as the ratio of these AUC: AUCd/AUCpp and a compliance index was also added as (distension/diastolic diameter)/(pulse pressure/diastolic pressure).

### 2.3. Blood Pressure and Blood Pressure Variability in Conscious Rats

Two more groups of SHR, one with L-NAME in drinking water as described above (n=9) and one without (n=8), were implanted with a standard telemetric device (TA11PA-C40, Data Sciences International, the Netherlands) with a pressure transducer under short-term anesthesia with isoflurane. Blood pressure signals were continuously recorded by data analysis software (Dataquest ART®, DSI). Systolic (SBP), diastolic (DBP), mean blood pressure (MBP), pulse pressure (PP), and heart rate (HR) were automatically detected beat to beat with analysis software ECG auto® (v3.0.0.18, EMKA, France) and averaged every minute.

Data were analyzed 1 day before L-NAME treatment and then at days 7, 14, 21, and 28 of treatment and similarly for the control SHR group. Reported values of SBP, DBP, MBP, and PP, represented the mean of individual measurements over the 24 h recording period. Short-term variability of HR, SBP, DBP, and PP was calculated over 1-minute periods over 24h Average Real Variability (ARV), an index used in clinical studies, considered as the most potent index for short-term BP variability, evaluates the variability between consecutive and validated readings [17], and was calculated by the following formula:

(1) $$\begin{array}{c} ARV=\frac{1}{\sum W}\overset{n}{\underset{k=1}{\sum }}W\times \left|\;BP_{k}-BP_{k-1}\;\right| \end{array}$$

where k ranges from 1 to n, w is the time interval between BP_k-1_ and BP_k_, (1 minute) and n is the number of BP readings in 24 hours (1440 values).

### 2.4. Samples for Ex Vivo Experiments

Urines were collected during 24 hours 3 days before the end of the protocol; proteinuria and the urinary ratio protein/creatinine were measured (ABX PENTRA C400).

At the end of the experiments the rats were euthanized via a lethal dose of sodium pentobarbital i.v. Rat nasorectal length was measured. The left ventricle and left kidney were weighed. Left ventricle and left kidney weight were normalized to their ratio to nasorectal length (mg/cm). Thoracic and abdominal aortas were cleaned and stored in 4 % formaldehyde.

### 2.5. Determination of Arterial Structure and Composition

Arterial structure was determined and quantified in 4% formaldehyde-fixed thoracic and abdominal aortas extracted from the rats used for hemodynamic measurements. For that purpose, a piece of about 5 mm long, corresponding to the same site used for ultrasound measurements for both TA and AA, was cut and embedded in paraffin. The tissue was then extracted from individual paraffin blocks and inserted into a preformed paraffin recipient block (Tissue-Tek Quick-Ray System, Sakura Finetek France). The finished block was then cut into 4 *μ*m thick sections and mounted on Superfrost plus slides and subjected to independent tests [10].

Media cross-sectional areas (MCSA) and scleroproteins quantifications were performed by morphological analysis after a Sirius red and a three-color staining protocol (Masson's trichrome) as previously described [7]. Elastin organization and structure were fully investigated through disarray quantification [10]. For that purpose, loss of parallel orientation and increased dispersion/randomness of fibers were investigated, and microscopic disruptions of elastin fibers were counted blindly in different microscope fields for each segment of aorta per rat at high magnification.

For immunohistochemical analyses of cell-matrix interactions, a fibronectin polyclonal antibody (ab2040, Millipore) was used. Integrins accumulation was quantified with *α*5 integrin (ab1928, Millipore). This analysis was completed with an anti-FAK antibody (Ab40794, Abcam), which recognized focal adhesion kinase [10].

Heat-mediated antigen retrieval was performed in EDTA buffer pH 9 in a water bath for 30 min. Immunostaining was performed on a Dako autostainer using a peroxidase-labeled polymer-based detection system (Envision plus, Dako) and diaminobenzidine as a chromogen. No specific staining was observed when primary antibody was omitted from the protocol (negative control). The distribution and quantification of staining were determined by computer-directed color analysis performed with the noncommercial image processing software Mesurin® [10, 18].

### 2.6. Statistical Analysis

All data were expressed as the mean ± the standard error of the mean (SEM). Then each hemodynamic parameter was analyzed with a one way ANOVA of raw data followed by a Tukey post-hoc comparison first at basal blood pressure and again at matched blood pressures across the three groups. Time effect was analyzed by a one WAY ANOVA followed by a Dunnett's test. Paired Student t test was performed to compare thoracic and abdominal values, as well as the effect of clonidine in hypertensive rats.

For immunochemistry analysis, two-way ANNOVA on groups and sites was performed followed by a Tukey's multiple comparison test.

Differences were considered significant at values of P<0.05.

## 3. Results

### 3.1. Evaluation of the Model: Effect of L-NAME Treatment in SHR

A slight reduction of body weight was observed in the SHRLN group. No groups presented with renal hypertrophy as determined by no change in animal-length normalized kidney weights. This was observed despite a reduction in kidney function in the SHRLN as determined by increased total proteinuria/24h and by the increased ratio urinary protein/creatinine (Table 1).

Left ventricular hypertrophy was observed in both hypertensive groups as determined by length-normalized left ventricle weight. The SHR had larger left ventricles compared to those of WKY and those of SHRLN were larger than those of SHR. The data are shown in Table 1.

**Figure 1:**
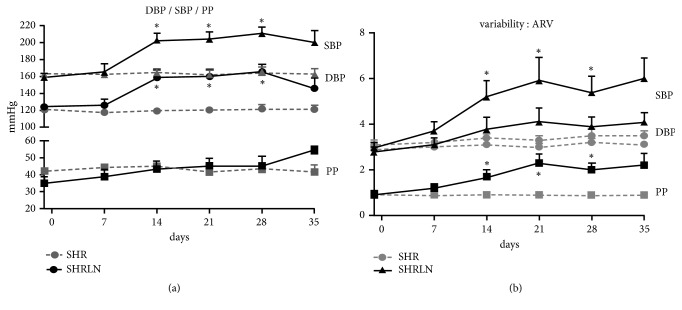
(a) time-evolution of systolic blood pressure (SBP, triangles), diastolic blood pressure (DBP, circles), and pulse pressure (PP, squares) in spontaneously hypertensive rat (SHR, grey symbols, and n=8) and in SHR during 5-week L-NAME treatment (SHRLN, black symbols, and n=9) and (b) short-term blood pressure variability as ARV (average real variability) are shown with similar symbols. *∗*: p<0.05 one way ANOVA and Dunnett's posttest on time effect.

The effect of L-NAME treatment on BP and short-term BP variability was evaluated in conscious rats used specifically for these measurements (n=8 SHR and n=9 SHRLN). L-NAME treatment increased BP in SHR. During the last week, 2 telemetered SHRLN died and others began to present slight decrease in blood pressure; thus only the 4 first weeks can be statistically analyzed. In SHR, SBP, DBP, PP, and their respective BPV were not modified during the 4 weeks. In SHRLN, SBP and DBP increased simultaneously with no change in PP. SBP BPV increased from day 14 with no change in DBP BPV, leading to a strong increase of PP BPV. (Figure 1)

### 3.2. Thoracic Aortic Stiffness Evaluation

**Figure 2:**
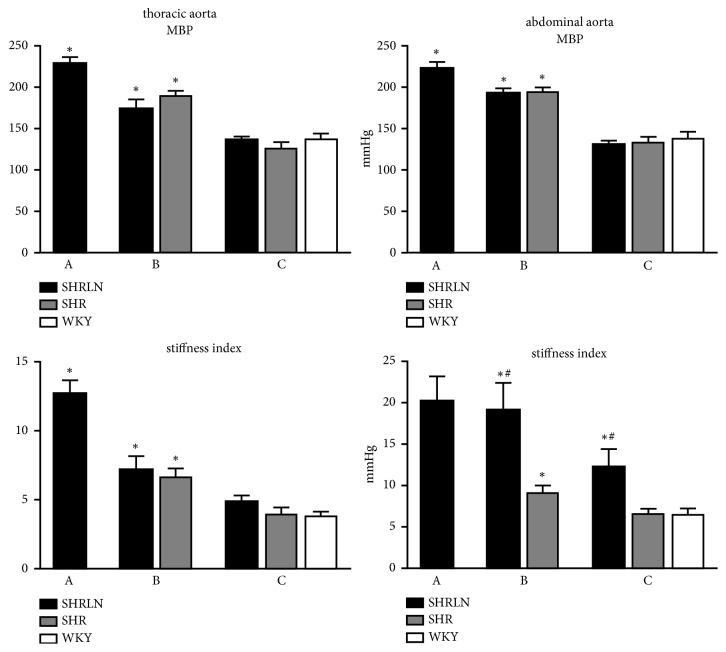
Mean blood pressure (MBP) and stiffness index are compared in the three groups of rats. Black bars: spontaneously hypertensive rats treated with L-NAME (SHRLN), grey bars: SHR without treatment, and white bars: normotensive WKY. Thoracic aorta (left graph) and abdominal aorta (right graphs) parameters are shown. A: SHRLN, parameters at basal pressure, and n= 8; B: SHRLN n=5, at BP matched with that of SHR n=6; C: both SHRLN n=7 and SHR n=6 at BP matched with that of WKY n=6. N are similar at TA and AA except n=5 for WKY at AA. One way ANOVA and Tukey posttest comparison: *∗*: p<0.05 compared to WKY and #: p<0.05 compared to SHR.

**Figure 3:**
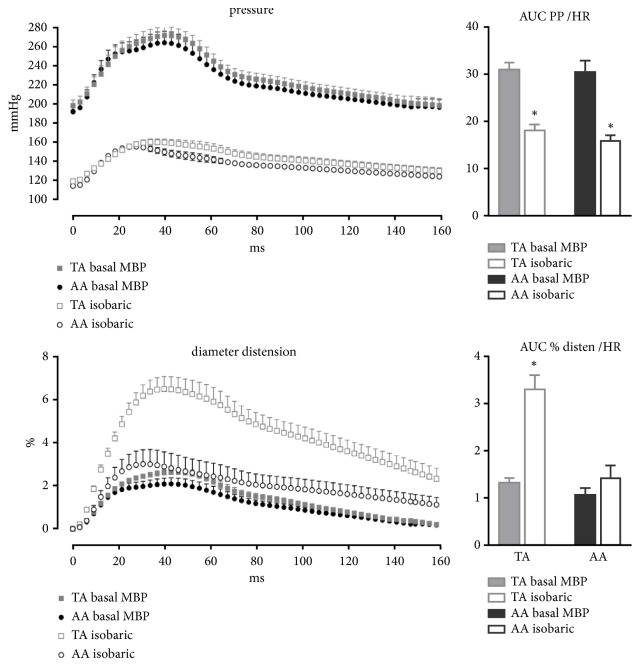
Changes in pressure and diameter over time emphasized the difference between thoracic aorta (TA) and abdominal aorta (AA) stiffening in hypertensive rats with reduced NO (SHRLN). The dynamic pressure wave and diameter distension waves throughout the cardiac cycle are shown for SHRLN TA and AA at basal mean blood pressure (MBP), n=7 and 8, respectively, and at reduced MBP (after clonidine administration), n=5 for each site. BP levels are similar for TA and AA whereas only TA distension recovers when BP decreases. Bars on the right quantify these data via measuring the area under the curve (AUC adjusted to heart rate). Despite similar pulse pressures, only the thoracic aorta is shown to increase when blood pressure is reduced. *∗*: isobaric value differs from basal value (P < 0.05).

Basal blood pressure (MBP, SBP, and DBP) and pulse pressure (PP) were higher in SHR versus WKY and higher in SHRLN than in the two other groups. A similar pattern was observed for mean and diastolic diameters. Aortic stiffness was higher in SHR compared to that of WKY and L-NAME treatment leading to a greater increase in stiffness in SHRLN compared to SHR. This was demonstrated by increased *β*-stiffness index and local PWV as well by decreased distensibility, compliance index and pulse diameter (Table 2). The AUC distension/ms and the distensibility index which takes into account not only the values acquired from systole and diastole but also the wave shapes of PP and distension also confirmed our observations on aortic stiffness (Figures 2 and 3).

Under isobaric conditions, i.e., after decreasing BP and PP to that of WKY by administration of clonidine in SHR and SHRLN, we no longer observed differences in stiffness in the thoracic aorta between our three groups, demonstrating that the stiffness increase was strongly pressure-dependent. Distension remained slightly reduced in SHRLN compared to SHR at matched blood pressures (Table 2).

### 3.3. Abdominal Aortic Stiffness Evaluation

The AA diameter was significantly smaller than the thoracic diameter in all groups. Also, all stiffness and distensibility parameters indicated significantly higher stiffness and lower compliance as expected at this more distal part of aorta.

The differences observed in the three groups at basal blood pressure were similar to those observed at the thoracic site: pressures, stiffness index, and local PWV were higher in the SHR compared to the WKY and more so in the SHRLN; distensibility, compliance index, and distension were lower in SHR than in WKY and lower in SHRLN compared to SHR.

However, in contrast to the thoracic aorta, after clonidine administration, the abdominal aorta of SHRLN remained stiffer under isobaric conditions (Table 3): stiffness index and local PWV were still significantly higher than those of WKY and SHR. Isobaric distensibility, distension, distensibility index, and compliance index all remained reduced in SHRLN, indicating that the intrinsic stiffness of the AA of SHRLN was specifically increased compared to SHR and WKY.

This increase in stiffness in the SHRLN AA but not in TA under isobaric conditions was also made apparent by comparing diameter distension at both sites and both pressures (Figure 3).

Furthermore, during clonidine administration, the higher stiffness and lower distensibility of the AA was maintained in SHRLN compared to SHR.

### 3.4. Distension and Blood Pressure Waves

Wave analysis, quantified by the AUC corrected for HR (Tables 2 and 3), confirmed the higher PP and lower distension in SHRLN in TA and AA at baseline operational BP. Then they showed that after reduction of BP via administration of clonidine the distension wave of AA in SHRLN remained lower than that in SHR at similar BP level and remained lower than that in SHR and WKY at BP similar to that in WKY. Importantly, this parameter indicated that this observation was not dependent on the reduction in heart rate after clonidine injection.

As for the other parameters, in contrast to AA, TA distension wave recovered and was no longer different from that in WKY after BP reduction. Again importantly the decrease in heart rate was similar at TA and AA levels and the AUC value corrected for heart rate.

The difference observed between AA and TA is emphasized by comparing BP and distension wave in SHRLN (Figure 3).

### 3.5. Arterial Structure and Composition

The vascular wall thickness did not significantly differ between SHR and WKY but was increased in SHRLN compared to that of WKY and SHR. These observations were similar for TA and AA. The internal diameter was comparable in the three groups for the two sites. As expected AA diameter was much lower than TA diameter (-33 % in WKY, -41 % in SHR, and -33 % in SHRLN), in agreement with* in vivo *data (respectively, -39 %, -41 %, and -41%).

**Figure 4:**
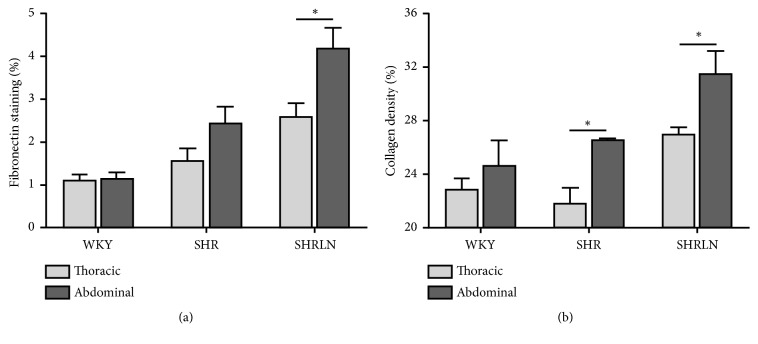
Comparison of fibronectin (a) and collagen (b) accumulation in the thoracic (TA) and abdominal (AA) aorta between SHRLN n=8, SHR n=6, and WKY n=6. The data show that AA levels of fibrosis markers were specifically higher in AA from SHRLN in agreement with stiffness data* in vivo*. Whilst collagen was also higher in AA in the SHR, these data show that fibronectin (Fn) content of the SHR AA remained similar to those of TA unlike the findings in the SHRLN. *∗*: p<0.05 AA versus TA in the same group of rats. Statistics between groups are shown in Table 3.

Immunohistochemical characteristics of the TA and AA structures appear in Table 4. Elastin density did not significantly differ between groups in both TA and AA. Collagen density as well as the ratio collagen/elastin and *α*5-integrin were increased only in SHRLN (for both TA and AA). Fibronectin and FAK were higher in SHR than in WKY and further increased in SHRLN compared to both WKY and SHR for the two sites. While there is a tendency for the SHR to show accumulation of fibrosis in the AA compared to the TA, we showed that it was only in the SHRLN that we had a strong specific accumulation of fibronectin and collagen at the AA and that this was associated with a significant increase in isobaric stiffness (Figure 4).

## 4. Discussion

There were two important findings in the present study: (1) the major effects of chronic NO reduction in hypertensive rats were characterized by a greater increase in BP and aortic stiffening associated with structural changes in the aortic wall; (2) a specific pressure independent increase in AA stiffness compared to TA in the SHRLN which is associated with greater changes in fibrotic markers.

Large artery stiffening is now recognized as a cardiovascular risk factor as these compliant arteries lose their capacity to dampen the pulsatile force of cardiac systolic ejection. Increased blood pressure reduces arterial compliance but long-term high blood pressure as well as aging and chronic kidney disease induces moreover vascular wall remodeling which in turn further increases arterial wall stiffening in a vicious circle. Vascular wall remodeling involves alteration of numerous vascular wall components: increases in collagenous and fibrotic components, calcium deposition, reduction, and/or fragmentation of elastin and alteration of the vascular smooth muscle cells and is closely related to alteration of the endothelial cells and NO bioavailability. These mechanisms are complex and still not entirely understood [19, 20]. There is therefore a need to develop better animal models and better techniques of investigation. It is also necessary to separate the contribution of operating pressure to a stiffness measurement and the stiffening due to the long term remodeling of the vascular wall. For this purpose, we analyze the pressure-dependent stiffening at high basal blood pressure in hypertensive rats and the remodeling-dependent stiffening via adjustment of blood pressure to that of normotensive rats.

Our previous study on SHR treated with the NO inhibitor L-NAME demonstrated a major role of endothelial dysfunction on the development of arterial stiffness. After just two weeks of treatment, the animals presented with increased BP, cardiac hypertrophy, renal dysfunction, increased BP variability, and severe aortic stiffening characterized by arterial remodeling. This severe hypertensive model presented similar features with both very old SHRs [14] and interestingly with pathologies seen in human aging.

This first model experienced a significant degree of morbidity; thus it seemed prudent to increase the duration and decrease the concentration of L-NAME administration to improve animal outcomes and to better evaluate its structural and mechanical effects on aorta. In the present study we reduced the dose of L-NAME and increased the duration of the treatment to five weeks. As in the short protocol, we obtained comparable increases in BP and BP variability in conscious rats. We observed end organ damage and increased heart weight (normalized via tibia length) in the SHR compared to WKY and a further increase with L-NAME treatment. Proteinuria and the protein/creatinine ratio were significantly increased in SHRLN, indicating renal dysfunction.

These parameters allowed us to conclude that the five-week protocol is relevant for further studies on the role of NO reduction in vascular pathology and will be useful for both investigating the development of structural versus dynamic changes in the aorta and investigating the effects of therapeutic treatments on vascular stiffness.

Among the characteristics of the SHRLN model, BPV presents two further interests. First, the significant increase in BPV confirms the increase of intrinsic stiffness found in SHRLN as discussed below. Indeed, while several mechanisms may account for the BPV, the ability of the aorta to effectively buffer the pulsatile cardiac output is certainly a key component in regulating fluctuations in BP [21].

Second, despite correlated systolic and diastolic BP elevation, systolic but not diastolic BPV was parallelly increased throughout the duration of the treatment leading to a strong increase in PP variability. A similar pattern was observed in the short protocol and seems specific to this animal model [15], possibly related to NO reduction. It should be evaluated in clinical studies to evaluate the relevance of PP variability in pathological conditions.

The primary objective of this study was to, for the first time in this model, investigate the mechanostructural relationship of both the AA and TA in the same rats. We observed that the TA presented pressure-dependent stiffening, therefore disappearing when BP matched that of normotensive rats, in contrast to AA which presented both a pressure-independent and a remodeling-dependent stiffening; this is to say that arterial stiffness remained increased under normotensive isobaric conditions. This was shown via the usual parameters of compliance, distensibility and stiffness index measured at maximal systolic PP and distension. These findings are demonstrated in Figure 3 via the distension wave measurement previously developed in our laboratory [13]. Moreover, in addition to decreasing BP in the two groups of hypertensive rats to match that of WKY, we made an additional measurement in SHRLN at a BP matched to that of SHR, for the first time here; these results helped to reinforce our conclusions.

We recently compared AA and TA in a different model of hypertensive rats treated with a high salt diet [10] and observed the same pattern of stiffness in TA and AA. Nevertheless, the aortic structural alterations were different in SHRSP-salt and SHRLN. We observed the same increase in fibronectin and its associated integrins with no change in collagen content both in the TA and AA of SHRSP-salt. Also, these proteins could not account for the higher specific pressure-independent stiffening of the AA [10]. A similar result was found in a calcification model, wherein collagen was similarly no higher in AA than TA despite higher stiffness [22]. The structural parameter associated with the* in vivo* difference in arterial stiffness between the two sites in SHRSP-salt was elastin disarray which was observed only in the AA. In contrast to the salt model we did not observe elastin disarray in AA in the present study but a greater increase in collagen in both sites as previously observed [15].

As in the salt model, accumulation of fibronectin, *α*5-integrin, and focal adhesions (FAK) were also present in SHRLN in the AA and TA. Nevertheless, we observed a strong specific accumulation in the AA of SHRLN which may help to account for the increase in isobaric stiffness. These findings indicate that these fibrotic tissues begin to have a major impact on intrinsic arterial stiffness when a threshold of total fibrosis is reached. Indeed, fibronectin and collagen densities in the TA of SHRLN were close to the values of AA in SHR (Figure 4) and not associated with increases in isobaric stiffness, indicating that slight increases in these proteins are not sufficient to induce arterial pressure-independent stiffening. In contrast, their specific higher accumulation at the abdominal level in SHRLN was associated with a significant increase in isobaric stiffness.

Fibrosis is generally considered as a major factor of remodeling and stiffening [23]. The conclusion of the present study is in line with this but raises the important issue of a necessary threshold to observe hemodynamic consequences. This may explain conflicting results existing in the literature. Fibrosis development could be a “silent” detrimental factor whose pathophysiological consequences are visible upon reaching this threshold. Further experiments might consider using aging hypertensive rats at different periods since aged SHR presented both high fibrosis and strong stiffening [14, 23] in order to determine if the threshold theory holds true. Another important issue to address in further experiments is to explore the role of other components of the extracellular matrix as well as their crosstalk with vascular smooth muscle cells [20].

Several points regarding our methodology should be addressed although they have been largely discussed in our previous publication [10]. First the use of clonidine, which has been validated and compared with other BP hypotensive agents, presents the advantage of the duration of action. Second, we do not calculate wall stress values because it mixes* in vivo* values, at two different BP levels, with* ex vivo* measures and cannot be matched* in vivo *between aortic sites or groups. In our last publication we nevertheless calculated wall stress values but this did not offer any additional conclusions. The major interest of our technical approach is to evaluate the dynamic properties of a precise segment of vessel in living animals and we have demonstrated the relevance of using an acute decrease in BP to obtain isobaric parameters in different groups of animals in order to differentiate the effect of the operating pressure on the vascular wall and the long-term structural remodeling [13].

The thoracic site used between the diaphragm and the renal artery is often included in the abdominal aorta despite having diameter and compliance properties different from those of the abdominal infrarenal aorta. This site could have been alternatively named as the suprarenal aorta and our AA site the infrarenal aorta. The diaphragm is the limit between the thorax and the abdomen and by the way is often shown in anatomic schemes, as the limit between TA and AA but there no evidence that it is the functional and structural aortic limit. The difference between TA and AA embryologic development has not been related to the diaphragm. In human the length of the suprarenal but infradiaphragm is consistent and mainly called as superior abdominal aorta or suprarenal aorta; however a reduced vasa-vasorum, high incidence of aortic aneurysms, and reduced elastin level are described specifically for the infrarenal aorta [24]. Prevalence of aneurysm is lower in the upper part of the descending aorta and even much lower at the suprarenal aorta. Thus the three entities differ in the descending aorta thoracic, suprarenal, and infra-renal abdominal aorta. In rats, the suprarenal but infradiaphragm part of the aorta is short and almost never studied. As in human the abdominal embryologic, structural and functional specificity are described for the infrarenal aorta [25]. A technical reason to study the site under the diaphragm is that our method allows a non-invasive measurement of aortic diameter and this level can be recorded without opening the thoracic cage and present good vascular landmarks which improve reproducibility. We had previously observed and confirm in the present study the huge difference in diameter between suprarenal and infrarenal (60% larger above) and compliance (x4 above) in agreement with the TA versus AA characteristics. Therefore, the renal circulation which is high and higher preserved is being fed by the most compliant TA. In our previous study [10] we aimed to confirm our hypothesis by taking a ring of upper TA, above the diaphragm and comparing via the histological staining, the diameters, and ratio thickness/lumen. The data showed a huge change in diameter between the AA and the supra-renal level, whereas there was only a small change between the suprarenal site and the thoracic above the diaphragm.

In conclusion, the data presented give evidence that NO reduction, in addition to hypertension, induces fibrosis which reaches a high level in the abdominal aorta leading to a remodeling-dependent arterial stiffening.

## Figures and Tables

**Table 1:** Parameters for the model characterization.

		WKY	SHR	SHRLN
Body weight	g	432 ± 3	398 ± 12	373 ± 15*∗*
Left ventricle weight	mg/cm	32.8 ± 0.4	45.2 ± 1.3*∗*	50.4 ± 1.0*∗*#
Kidney weight	mg/cm	53.8 ± 8	54.1 ± 1.6	56.7 ± 1.7
24h proteinuria	mg/24h	0.98 ± 0.09	9.50 ± 1.12	47.3 ± 12.5*∗*#
Protein/creatinine		0.65 ± 0.01	0.69 ± 0.07	3.96 ± 1.11*∗*#
n		6	6	8

Parameters measured to characterize the model confirm previous observations: the body weight was slightly reduced in SHRLN, kidney weight was not modified but 24h proteinuria and protein/creatinine ratio were increased indicating renal dysfunction. Left ventricular hypertrophy was visible in SHR but was more pronounced in SHRLN as shown by its normalized weight. Results are expressed as mean ± SEM. *∗*significantly different (P < 0.05) compared to WKY; #significantly different (P < 0.05) compared to SHR basal. The numbers of rats (n) are indicated at the bottom of the table.

**Table 2:** Thoracic aorta hemodynamics, diameter, and stiffness measurements.

Thoracic aorta	WKY	SHR basal	SHR	SHRLN basal	SHRLN	SHRLN
			BP/WKY		BP/SHR	BP/WKY
Mean AP mmHg	138 ± 6	190 ± 5*∗*	127 ± 6	229 ± 7*∗*#	175 ± 11*∗*	138 ± 3
Systolic AP mmHg	160 ± 6	223 ± 7*∗*	151 ± 7	274 ± 9*∗*#	203 ± 12*∗*	161 ± 3
Diastolic AP mmHg	122 ± 6	163 ± 4*∗*	107 ± 5	198 ± 6*∗*#	154 ± 11*∗*	119 ± 2
Heart rate bpm	380 ± 20	362 ± 14	269 ± 10*∗*	378 ± 11	306 ± 27	264 ± 19*∗*
Pulse pressure mmHg	38 ± 1	61 ± 4*∗*	44 ± 3	77 ± 4*∗*#	50 ± 2*∗*#	42 ± 2
Local PWV m/s	5.7 ± 0.1	8.9 ± 0.5*∗*	5.5 ± 0.4	13.6 ± 0.7*∗*#	8.9 ± 0.8*∗*	6.4 ± 0.3
Mean diameter *μ*m	2517 ± 68	2856 ± 49*∗*	2691 ± 75	3060 ± 51*∗*#	2897 ± 65*∗*	2853 ± 66*∗*
Diastolic diameter *μ*m	2390 ± 68	2756 ± 49*∗*	2499 ± 87	2981 ± 48*∗*#	2808 ± 65*∗*	2732 ± 76*∗*
Aortic distension *μ*m	176 ± 9	136 ± 8*∗*	231 ± 16*∗*	80 ± 5*∗*#	126 ± 19	179 ± 11#
Aortic distension %	7.4 ± 0.4	4.9 ± 0.3*∗*	9.3 ± 0.9	2.7 ± 0.1*∗*#	4.5 ± 0.7*∗*	6.6 ± 0.6#
Compliance 10^−3^mm^2^/kPa	140.3 ± 6.4	78.4 ± 8.1*∗*	174.7 ± 16.8	39.6 ± 4.9*∗*#	90.0 ± 15.6*∗*	145.8 ± 6.4
Distensibility 10^−3^/kPa	29.4 ± 1.1	12.7 ± 1.4*∗*	32.7 ± 4.3	5.4 ± 0.6*∗*#	14.3 ± 2.8*∗*	23.8 ± 2.1
Stiffness index	3.9 ± 0.2	6.7 ± 0.6*∗*	4.0 ± 0.4	12.8 ± 0.9*∗*#	7.3 ± 0.9*∗*#	4.9 ± 0.4
AUCp/ms: pulse pressure	16.3 ± 0.6	26.9 ± 1.5*∗*	20.3 ± 1.6	31.1 ± 1.4*∗*	20.6 ± 0.8*∗*#	18.2 ± 1.0
AUCd/ms 10^−1^: distension	3.7 ± 0.2	2.6 ± 0.2*∗*	5.0 ± 0.4	1.3 ± 0.1*∗*#	2.2 ± 0.4*∗*	3.3 ± 0.3#
distensibility index 10^−2^	22.9 ± 0.8	9.9 ± 1.0*∗*	25.3 ± 3.2	4.5 ± 0.5*∗*#	11.0 ± 2.1*∗*	18.4 ± 1.5
(AUCd/AUCp)						
Compliance index	23.9 ± 1.3	13.7 ± 1.3*∗*	23.5 ± 2.4	7.1 ± 0.6*∗*#	13.6 ± 1.9*∗*	19.1 ± 1.6
n	5	6	6	8	5	7

Parameters measured and calculated via the echotracking at the thoracic aorta. Results are expressed as mean ± SEM. *∗*significantly different (P < 0.05) compared to WKY; #significantly different (P < 0.05) compared to SHR basal. The numbers of rats (n) are indicated at the bottom of the table.

**Table 3:** Abdominal aorta hemodynamics, diameter, and stiffness measurements.

Abdominal aorta	WKY	SHR basal	SHR	SHRLN basal	SHRLN	SHRLN
			BP/WKY		BP/SHR	BP/WKY
Mean AP mmHg	139 ± 7	196 ± 4*∗*	134 ± 5	223 ± 8*∗*#	195 ± 4*∗*	133 ± 2
Systolic AP mmHg	164 ± 7	233 ± 6*∗*	160 ± 5	268 ± 11*∗*#	230 ± 6*∗*	157 ± 2
Diastolic AP mmHg	122 ± 7	166 ± 3*∗*	113 ± 5	192 ± 6*∗*#	169 ± 4*∗*	116 ± 2
Heart rate bpm	387 ± 15	368 ± 13	285 ± 7*∗*	368 ± 8	334 ± 20	270 ± 18*∗*
Pulse pressure mmHg	42 ± 1	66 ± 4*∗*	47 ± 2	77 ± 5*∗*	60 ± 3*∗*	41 ± 2#
Local PWV m/s	7.6 ± 0.4	10.6 ± 0.5*∗*	7.5 ± 0.4	16.4 ± 1.2*∗*#	15.1 ± 1.1*∗*#	10.0 ± 0.8*∗*#
Mean diameter *μ*m	1529 ± 45	1695 ± 17*∗*	1616 ± 53	1799 ± 34*∗*	1744 ± 42*∗*	1738 ± 39*∗*
Diastolic diameter *μ*m	1478 ± 45	1630 ± 22*∗*	1543 ± 58	1698 ± 34*∗*#	1665 ± 59*∗*	1675 ± 46
Aortic distension *μ*m	72 ± 8	62 ± 4	88 ± 9	37 ± 5*∗*#	31 ± 4*∗*#	49 ± 8#
Aortic distension %	4.9 ± 0.6	3.8 ± 0.3	5.7 ± 0.6	2.2 ± 0.3*∗*#	1.8 ± 0.3*∗*#	3.0 ± 0.5#
Compliance 10^−3^mm^2^/kPa	30.7 ± 3.3	19.1 ± 1.7*∗*	36.3 ± 4.6	10.3 ± 1.3*∗*#	10.5 ± 1.4*∗*#	24.1 ± 3.0#
Distensibility 10^−3^/kPa	17.4 ± 1.6	8.7 ± 0.8*∗*	18.3 ± 2.2	4.3 ± 0.6*∗*#	4.6 ± 0.7*∗*#	10.8 ± 1.7#
Stiffness index	6.6 ± 0.7	9.3 ± 0.8*∗*	6.7 ± 0.5	20.4 ± 2.9*∗*#	19.2 ± 3.2*∗*#	12.5 ± 2.0*∗*#
AUCp/ms: pulse pressure	16.7 ± 0.6	29.0 ± 1.6*∗*	20.8 ± 1.1*∗*	30.5 ± 2.3*∗*	24.2 ± 1.8*∗*	16.0 ± 1.0#
AUCd/ms 10^−1^: distension	2.3 ± 0.3	1.9 ± 0.1	2.9 ± 0.3	1.1 ± 0.1*∗*#	0.9 ± 0.1*∗*#	1.4 ± 0.3#
distensibility index 10^−2^	13.4 ± 1.2	6.7 ± 0.6*∗*	14.4 ± 1.8	3.6 ± 0.5*∗*#	3.9 ± 0.6*∗*#	8.7 ± 1.3#
(AUCd/AUCp)						
Compliance index	14.0 ± 1.0	9.8 ± 0.9*∗*	13.7 ± 1.2	5.7 ± 0.8*∗*#	5.3 ± 0.9*∗*#	8.5 ± 1.3*∗*#
n	5	6	6	8	5	7

Parameters measured and calculated via the echotracking at the thoracic aorta. Results are expressed as mean ± SEM. *∗*significantly different (P < 0.05) compared to WKY; #significantly different (P < 0.05) compared to SHR basal. The numbers of rats (n) are indicated at the bottom of the table.

**Table 4:** Aortic structure and composition.

Aortic site		Thoracic			Abdominal	
Group	WKY	SHR	SHRLN	WKY	SHR	SHRLN
Thickness *μ*m	114 ± 2	129 ± 2	152 ± 82^*∗*#^	105 ± 3	111 ± 5	131 ± 4^*∗*#^
Lumen *μ*m	1334 ± 86	1541 ± 36	1498 ± 86	888 ± 39	912 ± 44	1002 ± 38
MCSA, AU	950 ± 36	1017 ± 14	1059 ± 44	520 ± 19	648 ± 11	752 ± 56*∗*
MCSA /BW, mg/AU	6.76 ± 0.26	7.77 ± 0.16*∗*	9.00 ± 0.9*∗*	4.28 ± 0.63	4.95 ± 0.82	6.30 ± 0.65^*∗*#^
Elastic Lamellae, n	9.7 ± 0.9	8.0 ± 0.5	8.1 ± 0.4	7.3 ± 0.4	7.2 ± 0.2	7.4 ± 0.3
Interlamellar Space, AU	0.97 ± 0.04	1.14 ± 0.06*∗*	1.16 ± 0.08*∗*	0.65 ± 0.04	0.62 ± 0.04	0.64 ± 0.04
Colored Aortic Wall:						
(i) VSMC nucleus, %	7.0 ± 0.6	7.2 ± 0.4	9.6 ± 0.6^*∗*#^	7.4 ± 0.5	7.7 ± 0.7	11.5 ± 1.1^*∗*#^
(ii) Collagen density %	22.9 ± 0.8	21.9 ± 1.1	27.0 ± 0.5^*∗*#^	24.7 ± 1.8	26.6 ± 0.1	31.5 ± 1.7^*∗*#^
(iii) Elastin density %	21.2 ± 1.5	21.8 ± 2.1	18.6 ± 1.0	13.7 ± 0.6	12.7 ± 0.7	13.0 ± 0.6
(iv) Collagen/elastin	1.08 ± 0.07	1.03 ± 0.09	1.47 ± 0.06^*∗*#^	1.81 ± 0.15	2.03 ± 0.09	2.42 ± 0.16^*∗*#^
Stained Aortic Wall, %:						
(i) Fibronectin	1.11 ± 0.13	1.58 ± 0.26	2.60 ± 0.31^*∗*#^	1.15 ± 0.13	2.45 ± 0.36*∗*	4.20 ± 0.46^*∗*#^
(ii) *α*5-integrin	2.61 ± 0.44	3.81 ± 0.51	4.64 ± 0.31*∗*	2.92 ± 0.36	4.04 ± 0.21*∗*	5.20 ± 0.17^*∗*#^
(iii) FAK	3.28 ± 0.59	4.93 ± 0.48*∗*	9.12 ± 0.09^*∗*#^	1.11 ± 0.58	5.28 ± 0.24*∗*	8.71 ± 0.22^*∗*#^
n	6	6	8	6	6	8

Results are expressed in percentage of colored or stained media area and reported as mean ± SEM. *n* = 6-8 rats per group. MCSA: media cross sectional area. BW: Body weight. FAK: focal adhesion kinase

*∗*significantly different (P < 0.05) compared to WKY; #significantly different (P < 0.05) compared to SHR. The numbers of rats (n) are indicated at the bottom of the table.

## Data Availability

The data used to support the findings of this study are included within the article. The detailed data used to support the findings of this study are available from the corresponding author upon request.

## Abbreviations

SHRLN: Spontaneously hypertensive rat treated with L-Name
AA: Abdominal aorta
TA: Thoracic aorta
PP: Pulse pressure
NO: Nitric oxide
BP: Blood pressure
SBP: Systolic blood pressure
DBP: Diastolic blood pressure
MBP: Mean blood pressure
HR: Heart rate
BPV: Blood pressure variability
FAK: Focal adhesion kinase.
